# Editorial: Structure and function of the immunological and redirecting artificial synapses and their clinical implications

**DOI:** 10.3389/fimmu.2024.1497118

**Published:** 2024-10-03

**Authors:** Manuel Izquierdo, Javier Ruiz-Navarro, Cosima T. Baldari, Pedro Roda-Navarro

**Affiliations:** ^1^ Instituto de Investigaciones Biomédicas Sols-Morreale (IIBM), Consejo Superior de Investigaciones Científicas-Universidad Autónoma de Madrid (CSIC-UAM), Madrid, Spain; ^2^ Translational Research in Pediatric Oncology, Hematopoietic Transplantation and Cell Therapy, Instituto de Investigación Sanitaria del Hospital Universitario La Paz (IdiPAZ), Madrid, Spain; ^3^ Department of Life Sciences, University of Siena, Siena, Italy; ^4^ Department of Immunology, Ophthalmology and ear, nose and throat (ENT), School of Medicine, Universidad Complutense, Madrid, Spain; ^5^ Lymphocyte Immunobiology Group, Instituto de Investigación Sanitaria12 de Octubre (imas12), Madrid, Spain

**Keywords:** immune synapse, T lymphocytes, chimeric antigen receptor (CAR), actin cytoskeleton, T cell-redirecting strategies, secretory traffic

The immune system comprises a compendium of specialized cells, tissues, and organs that protect the organisms from a myriad of pathogens and can survey and destroy cancer cells. The recognition of the cognate antigens by the T cell and B cell receptor (TCR and BCR), which is required for the activation of antigen-specific effector functions of T and B lymphocytes is executed in the context of the immunological synapse (IS). The IS is a specialized cell-cell interaction that acts as a signaling platform for the integration of signals leading to intercellular communication, in order to ensure efficient TCR and BCR-evoked signal transduction.

IS formation triggers the asymmetric reorganization of the actin and tubulin cytoskeleton and the convergence of secretory vesicles, including multivesicular bodies (MVBs) (1, 2) and other lysosome-related organelles (LROs), toward the microtubule-organizing center (MTOC), which polarizes along with the secretory vesicles to the IS (3). These traffic events lead to the exocytosis of LROs and cytokine-containing vesicles at the synaptic cleft. LRO content secreted at the IS (including perforin, granzymes and intraluminal vesicles released as exosomes) plays an important role in cytotoxic T lymphocyte (CTL) mediated cytotoxicity (3), activation-induced cell death (4), germinal center reaction, antibody production, and antigen extraction at the B lymphocyte IS (5), whereas cytokine secretion plays an important role in antigen-presenting cell (APC) stimulation (6).

In the context of antigen recognition, MVB degranulation and extracellular vesicle (EV) secretion, we have received a relevant review on microvilli and EVs at the IS (Ruiz-Navarro et al.). This contribution addresses the different classes of EVs detected at the IS, emphasizing the most recent findings on microvilli/lamellipodium-produced EVs. Microvilli connections are F-actin-rich protrusions that act as sensors involved in the palpation of antigen-presenting cells and antigen sensing and are fundamental initiators of IS assembly (**Figure 1**); however, recent evidence has shown an important role of microvilli not only in antigen sensing but also in effector functions by producing EVs. The signals that lead to the polarized secretion of EVs at the synaptic cleft are discussed in this study, showing that the IS architecture fulfills a fundamental task in this secretion route.

**Figure 1:**
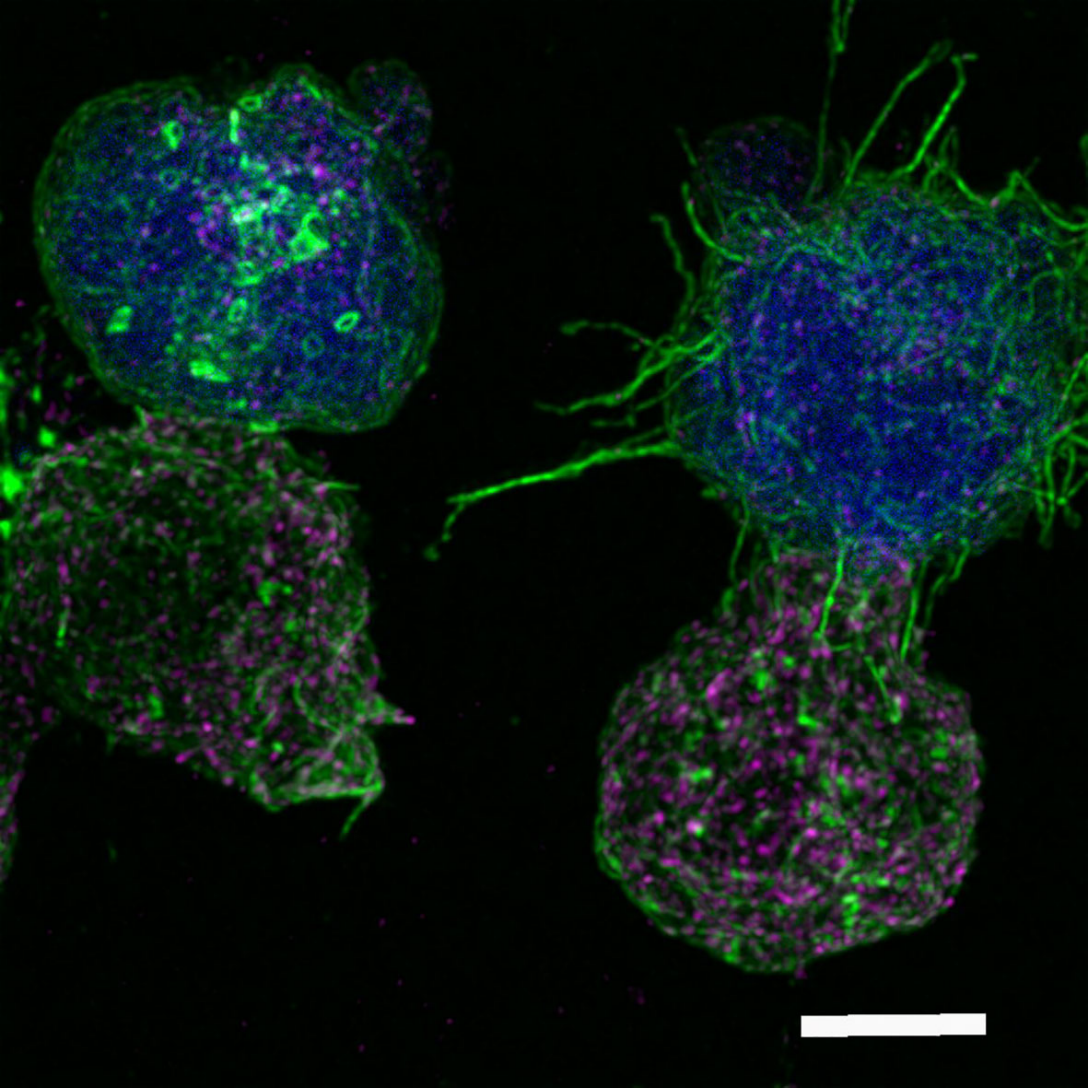
Immunological synapse connection. Human Jurkat T lymphocytes conjugated to the superantigen staphylococcal enterotoxin E pulsed Raji cells previously labeled with cell tracker (blue). After 1 h of synaptic conjugate formation on chambered coverslips, fixed cells were labeled with phalloidin-AF488 (green) and anti-CD63-AF633, to visualize F-actin and multivesicular bodies (magenta), respectively, and imaged by confocal fluorescence microscopy. Optical sections were acquired every 0.3 microm and maximal intensity projections were generated. The image shows two Jurkat T lymphocytes (bottom), forming synaptic conjugates (immunological synapse) with the antigen-presenting cells presenting the superantigen (top, blue). Filamentous actin appears green, whereas multivesicular bodies are the magenta vesicles. The cell conjugate on the left represents a mature synapse, whereas the conjugate on the right consists of an emerging synapse, showing the microvilli connections between the two cells. Microvilli connections are F-actin-rich protrusions that act as sensors involved in palpating the antigen-presenting cells and in antigen sensing and are fundamental initiators of the immune synapse (7). White bar = 5 µm. Authors: Manuel Izquierdo and Javier Ruiz-Navarro.

While significant attention has been focused on the T, B lymphocyte, and NK cell side of the IS, particularly on its role as a secretory platform for polarized secretion of diverse secretory vesicles (7), much less is known about the trafficking events occurring at the tumor cell side of the IS. Recent evidence supports the notion that tumor cells and APCs can rapidly reorganize their actin and tubulin cytoskeleton toward CTLs (8), Th cells (9) and NK cells (10). This reorganization underlies the polarized secretion of tumor cell LROs toward CTLs and NK cells, providing mechanisms that allow the tumor cell to evade the immune system (11). These results indicate that a view of IS based exclusively on analyses from the effector cell side of the synaptic cleft may only provide a partial view of the complex structural and functional interrelationship between the effector and the tumor cell. Indeed, a more holistic approach, which should include the study of the events occurring on the tumor cell/APC side of the IS is needed to achieve a global picture of IS structure and function. In this context, we have received an interesting contribution (Biolato et al.) on the reorganization of the F-actin cytoskeleton in human breast adenocarcinoma cells and melanoma cells forming IS with human NK cells. The authors, using elegant super-resolution imaging approaches based on a pH-sensitive probe for CD63^+^ vesicle exocytosis, suggest that directed secretion may occur from tumor cells toward attacking NK cells. Their findings reveal that synaptic remodeling of the actin cytoskeleton within cancer cells interacting with NK cells is associated with increased recruitment and fusion of MVBs at the IS, as was shown previously on the T lymphocyte side of the IS (1, 2) (Ruiz-Navarro et al.). This suggests that cancer cell-derived exosomes may be released into the IS, raising important questions about the properties of these exosomes and their potential role in conferring resistance to NK cell-mediated cytotoxicity. Although further investigation is needed to formally demonstrate IS-triggered exosome secretion by the tumor cells, and to reveal both the mechanism involved and the consequences of MVB secretory traffic, this contribution highlights a potentially overlooked mechanism by which cancer cells may evade immune destruction.

Regarding the translational implications that may be derived from applying such a holistic approach to IS studies, T-cell redirecting approaches to selectively eliminate tumor cells are useful strategies for cancer immunotherapy and are based on the formation of T cell-target cell conjugates (12), although these approaches have poor therapeutic results against solid tumors. These approaches include strategies to conjugate T lymphocytes with cancer cells using chimeric antigen receptors (CARs) (13, 14) or bispecific antibodies recognizing cell-surface tumor-associated antigens (15). Despite the fact that these two strategies promote the formation of functional ISs in terms of signals leading to LRO secretory polarized traffic and tumor cell death, the spatiotemporal architecture of the IS formed under these “artificial” redirecting strategies is different from that found in canonical, TCR-evoked synapses (12, 16). Canonical IS-tuned CAR signaling enhances the anti-tumor activity of CAR T cells and T-cell engaging bispecific antibodies (16) (17), and recent evidence shows that IS quality measurements correlate with patient clinical outcomes (18). Thus, the IS studies evoked during these approaches will indeed provide future experimental strategies to tune these “artificial” IS responses to improve T-cell effector anti-tumor responses (17).

Among the most common complications in CAR-T therapy are the failure to produce autologous CAR-T cells, the loss of target antigen, the barriers imposed by the tumor microenvironment, and the lack of efficacy and persistence (exhaustion) of CAR-T cells, which may eventually lead to tumor cell evasion by affecting the therapeutic CAR-T effect (15). In this context, we have received an interesting contribution from the NEXT Generation CART MAD Consortium (NEXT CART) (Martín-Antonio et al.), which is based on various basic and translational research groups and hospitals in Madrid (Spain) that have joined forces to share and synergize their basic expertise in IS, immunotherapy, gene therapy and their clinical expertise in pediatric and adult oncology. The aim of NEXT CART is to develop new cell engineering approaches and treatments for adult and pediatric neoplasms to be evaluated in multicenter clinical trials. The Consortium claims that “we will provide our perspective on how these strategies will evolve in the coming years to solve the main current limitations of these therapies”. Opportunities for advancement identified by the authors include developing allogeneic products, optimizing CAR signaling domains, combining cellular immunotherapies, multi-targeting strategies, and improving tumor-infiltrating lymphocyte (TIL)/TCR therapy.

Last but not least, with respect to IS-induced cell asymmetry, Gómez-Morón et al. submitted a relevant contribution showing how translation inhibition affects the asymmetric reorganization that occurs during CTL activation. The authors demonstrate that cytosolic protein translation is required to increase glucose metabolism and degranulation capacity upon TCR activation and thus regulate the full effector function of human CTLs. Interestingly, they observe that inhibition of translation shortly before TCR activation prevents proper reorganization of the tubulin cytoskeleton and asymmetric distribution of mitochondria. mTOR pathways are defectively activated in these cells and glucose metabolism through glycolysis and oxidative phosphorylation (OXPHOS) is affected. In this context, the movement of lytic granules (a type of LRO characteristic of CTLs) at the cell contact area with stimulating surfaces and the degranulation of CTLs are inhibited. Thus, the authors have linked the activity of 80S ribosomes and mitochondria to the ability of human CTLs to exert their effector functions *in vitro*.

Overall, our greatest reward has been to receive such relevant contributions, even after the deadline of the submission deadline, and to observe that the number of manuscript downloads and views is continuously increasing. We believe that thanks to the contributions included in this Research Topic, we have fulfilled our objective of editing a collection of articles that stimulates and facilitates a scientific forum for immunologists but also for clinicians, bridging the cross-disciplinary gap, increasing awareness, and maximizing the discussion and dissemination of ideas and methodologies in this emerging field.

## References

[1] Alonso R Mazzeo C Rodriguez MC Marsh M Fraile-Ramos A Calvo V. Diacylglycerol kinase alpha regulates the formation and polarisation of mature multivesicular bodies involved in the secretion of Fas ligand-containing exosomes in T lymphocytes. Cell Death differentiation. (2011) 18:1161–73. doi: 10.1038/cdd.2010.184 PMC3131963 21252909

[2] Calvo V Izquierdo M. Inducible polarized secretion of exosomes in T and B lymphocytes. Int J Mol Sci. (2020) 21:2631. doi: 10.3390/ijms21072631 32290050 PMC7177964

[3] Cassioli C Baldari CT. The expanding arsenal of cytotoxic T cells. Front Immunol. (2022) 13:883010. doi: 10.3389/fimmu.2022.883010 35514977 PMC9065447

[4] Alonso R Rodriguez MC Pindado J Merino E Merida I Izquierdo M. Diacylglycerol kinase alpha regulates the secretion of lethal exosomes bearing Fas ligand during activation-induced cell death of T lymphocytes. J Biol Chem. (2005) 280:28439–50. doi: 10.1074/jbc.M501112200 15870081

[5] Yuseff MI Pierobon P Reversat A Lennon-Dumenil AM. How B cells capture, process and present antigens: a crucial role for cell polarity. Nat Rev Immunol. (2013) 13:475–86. doi: 10.1038/nri3469 23797063

[6] Dustin ML Choudhuri K. Signaling and polarized communication across the T cell immunological synapse. Annu Rev Cell Dev Biol. (2016) 32:303–25. doi: 10.1146/annurev-cellbio-100814-125330 27501450

[7] Calvo V Izquierdo M. Role of actin cytoskeleton reorganization in polarized secretory traffic at the immunological synapse. Front Cell Dev Biol. (2021) 9:629097. doi: 10.3389/fcell.2021.629097 33614660 PMC7890359

[8] McKenzie B Khazen R Valitutti S. Greek fire, poison arrows, and scorpion bombs: how tumor cells defend against the siege weapons of cytotoxic T lymphocytes. Front Immunol. (2022) 13:894306. doi: 10.3389/fimmu.2022.894306 35592329 PMC9110820

[9] Ibanez-Vega J Del Valle Batalla F Saez JJ Soza A Yuseff MI. Proteasome dependent actin remodeling facilitates antigen extraction at the immune synapse of B cells. Front Immunol. (2019) 10:225. doi: 10.3389/fimmu.2019.00225 30873155 PMC6401660

[10] Ockfen E Filali L Pereira Fernandes D Hoffmann C Thomas C. Actin cytoskeleton remodeling at the cancer cell side of the immunological synapse: good, bad, or both? Front Immunol. (2023) 14:1276602. doi: 10.3389/fimmu.2023.1276602 37869010 PMC10585106

[11] McKenzie B Valitutti S. Resisting T cell attack: tumor-cell-intrinsic defense and reparation mechanisms. Trends cancer. (2023) 9:198–211. doi: 10.1016/j.trecan.2022.12.003 36593148

[12] Roda-Navarro P Álvarez-Vallina L. Understanding the spatial topology of artificial immunological synapses assembled in T cell-redirecting strategies: A major issue in cancer immunotherapy. Front Cell Dev Biol. (2020) 7. doi: 10.3389/fcell.2019.00370 PMC6965029 31998721

[13] Dagher OK Posey AD. Forks in the road for CAR T and CAR NK cell cancer therapies. Nat Immunol. (2023) 24:1994–2007. doi: 10.1038/s41590-023-01659-y 38012406 PMC12798859

[14] Baker DJ Arany Z Baur JA Epstein JA June CH. CAR T therapy beyond cancer: the evolution of a living drug. Nature. (2023) 619:707–15. doi: 10.1038/s41586-023-06243-w PMC12522170 37495877

[15] Blanco B Compte M Lykkemark S Sanz L Alvarez-Vallina L. T cell-redirecting strategies to 'STAb' Tumors: beyond CARs and bispecific antibodies. Trends Immunol. (2019) 40:243–57. doi: 10.1016/j.it.2019.01.008 30827461

[16] Davenport AJ Cross RS Watson KA Liao Y Shi W Prince HM. Chimeric antigen receptor T cells form nonclassical and potent immune synapses driving rapid cytotoxicity. Proc Natl Acad Sci U S A. (2018) 115:E2068–E76. doi: 10.1073/pnas.1716266115 PMC5834689 29440406

[17] Chockley PJ Ibanez-Vega J Krenciute G Talbot LJ Gottschalk S. Synapse-tuned CARs enhance immune cell anti-tumor activity. Nat Biotechnol. (2023) 41:1434–45. doi: 10.1038/s41587-022-01650-2 PMC10394118 36732477

[18] Naghizadeh A Tsao W-c Hyun Cho J Xu H Mohamed M Li D. *In vitro* machine learning-based CAR T immunological synapse quality measurements correlate with patient clinical outcomes. PloS Comput Biol. (2022) 18:e1009883. doi: 10.1371/journal.pcbi.1009883 35303007 PMC8955962

